# TRAIL inhibits angiogenesis stimulated by VEGF expression in human glioblastoma cells

**DOI:** 10.1038/sj.bjc.6603092

**Published:** 2006-04-11

**Authors:** G Cantarella, N Risuglia, R Dell'eva, L Lempereur, A Albini, G Pennisi, G M Scoto, D N Noonan, R Bernardini

**Affiliations:** 1 Department of Experimental and Clinical Pharmacology, University of Catania, Viale Andrea Doria, 6, Catania 95125, Italy; 2 Laboratory of Experimental Oncology, National Cancer Research Institute, Genova 16100, Italy; 3 Department of Chemical Sciences, University of Catania, Catania 95125, Italy; 4 Department of Pharmaceutical Sciences, University of Catania, Catania 95125, Italy

**Keywords:** brain tumour, endothelial cell, tissue remodelling, invasiveness

## Abstract

Tumour growth is tightly related to new blood vessel formation, tissue remodelling and invasiveness capacity. A number of tissular factors fuel the growth of glioblastoma multiforme, the most aggressive brain neoplasm. In fact, gene array analyses demonstrated that the proapoptotic cytokine tumour necrosis factor-related apoptosis-inducing ligand (TRAIL) inhibited mRNA expression of VEGF, along with those of matrix metalloproteinase-2 (MMP-2), its inhibitor tissue inhibitor of matrix metalloproteinases-2 (TIMP-2), as well as the tumour invasiveness-related gene secreted protein acid rich in cysteine (SPARC) in different human glioblastoma cell lines. Particularly, VEGF mRNA and protein expression and release from glioblastoma cells were also inhibited by TRAIL. The latter also exerted antimitogenic effects on human umbilical vein endothelial cells (HUVECs). With the same cells, TRAIL inhibited new vessel formation in the *in vitro* matrigel model, as well as it exerted powerful inhibition of blood vessel formation induced by an angiogenic cocktail administered in subcutaneous pellets *in vivo* in the C57 mouse. Moreover, the expression of MMP-2, its inhibitor TIMP-2 and the tumour invasiveness-related protein SPARC were effectively inhibited by TRAIL in glioblastoma cell lines. In conclusion, our data indicate that TRAIL inhibits the orchestra of factors contributing to glioblastoma biological aggressiveness. Thus, the TRAIL system could be regarded as a molecular target to exploit for innovative therapy of this type of tumour.

Formation of new blood vessels starting from pre-existing vascular structures is referred to as angiogenesis (Bussolino *et al*, 1998). Angiogenesis is dependent on an array of intrinsic stimulatory and inhibitory factors, interplaying among them with the aim to modulate both tissue matrix and cells participating in the process (Risau, 1997).

Vascular endothelial growth factor (VEGF) is the most specific among proangiogenic factors (Ferrara, 1993), whose expression is typically increased in normal and tumour cells at conditions of hypoxia (Ferrara and Davis Smyth, 1997). A quite large number of tumours are able to synthesise and actively release VEGF (Ferrara, 2005). Although of relevance, VEGF is not the sole factor concurring to angiogenesis. In fact, an array of regulatory molecules contributes substantially to the formation of new vessels (Folkman, 1995). For example, the proinflammatory macrophagic cytokine tumour necrosis factor-*α* (TNF-*α*) possesses direct proangiogenic properties (Vanderslice *et al*, 1998), and the TNF-related apoptosis-inducing ligand (TRAIL) has been shown to affect the function of endothelial cells (Li *et al*, 2003).

TRAIL binds the two transmembrane, homotrimeric DR4 and DR5 death receptors, as well as two decoy receptors and osteoprotegerin, involved in bone tissue remodelling (Almasan and Ashkenazi, 2003). Although biological effects of TRAIL were initially thought to be restricted to its typical tumour cell toxicity (Sheridan *et al*, 1997), numerous data report its proapoptotic effects on normal human cells, including brain cells (Nitsch *et al*, 2000). Interestingly, human glioblastoma cells, such as A172, U87MG and U373MG, display differential responsiveness to TRAIL-related cytotoxicity *in vitro* and *in vivo* (Pollack *et al*, 2001; Nabors *et al*, 2003; Kasuga *et al*, 2004), and also release substantial amounts of VEGF in response to hypoxia (Acker and Plate, 2004). Moreover, such a high angiogenic potential of glioblastoma appears associated with high expression of tissue remodelling-related molecules, such as the matrix metalloproteinase-2 (MMP-2) and its inhibitor, named tissue inhibitor of metalloproteinases-2 (TIMP-2), both concurring, along with angiogenesis, to tumour growth (Fillmore *et al*, 2001; VanMeter *et al*, 2001). Furthermore, glioblastoma growth is marked by severe dysfunction of molecules such as the secreted protein acid rich in cysteine (SPARC), which, in normal cells, restraints invasiveness and is actually being regarded as a candidate main enhancer of high tissue invasiveness (Golembieski and Rempel, 2002; Schultz *et al*, 2002).

Thus, in light of the reported effects of TRAIL on both tumour and endothelial cells, considering the high vascularisation index of glioblastomas (De Ridder *et al*, 1987), we first investigated the possible involvement of TRAIL in glioblastoma-related angiogenesis.

To do so, we verified the hypothesis whether TRAIL could influence angiogenesis-related genes in both the three human glioblastoma cell lines A172, U87MG and U373MG, as well as in human umbilical vein endothelial cells (HUVECs), by means of a specific microarray (Zheng *et al*, 2000). In parallel, we also evaluated direct effects of TRAIL on endothelial cell function.

To verify the hypothesis that factors modulating angiogenesis might, in addition, influence tissue factors related to tumour progression, we studied TRAIL effects upon tissue remodelling and invasiveness gene expression in different glioblastoma cell lines.

## MATERIALS AND METHODS

### Cell cultures and reagents

All materials and media were from Invitrogen Srl (San Giuliano Milanese, Italy), unless otherwise specified.

The human glioblastoma cell lines A172, U87MG and U373MG, as well as both the HeLa and the MCF-7 cell lines, were routinely cultured in Dulbecco's modified Eagle's medium (DMEM) supplemented with 10% (v v^−1^) fetal bovine serum (FBS), 50 *μ*g ml^−1^ penicillin and 100 *μ*g ml^−1^ streptomycin, and kept at 37°C in humidified 5% CO_2_/95% atmosphere.

HUVECs were obtained from human umbilical cords of healthy women undergone uncomplicated term pregnancies, as described elsewhere (Jaffe *et al*, 1973). HUVECs were grown in gelatin-coated plastic in medium M199 supplemented with endothelial cell growth supplement (ECGS; 20 *μ*g ml^−1^), heparin (1625 UI ml^−1^) (Sigma-Aldrich, Milano, Italy) and 20% FBS.

### Gene array

Human Angiogenesis GEArray kit (SuperArray Inc., Bethesda, MD, USA), composed of 96 angiogenesis-related genes as well as two housekeeping genes, actin and glyceraldehyde-3-phosphate dehydrogenase (GADPH), was used to characterise gene expression profile of A172 cells after treatment with TRAIL 25 ng ml^−1^ (Alexis Biochemicals, San Diego, CA, USA) for 0, 6, 12 and 24 h. Total RNA was isolated from cells after solubilisation in guanidinium thiocyanate by phenol–chloroform extraction and precipitation. cDNA probes for array analysis were synthesised following the manufacturer's directions. Differential gene expression patterns were detected by autoradiography. The experiments were repeated three times.

### Probe synthesis and Northern blot analysis

The cDNA probes of the VEGF gene and the GADPH were synthesised by RT–PCR. For the human VEGF, we used a primer pair composed of the sense primer 5′-TTGCTGCTCTACCTCCAC-3′ and the antisense primer 5′-AATGCTTTCTCCGCTCTG-3′; for the human GADPH, the primer set was composed of the sense 5′-CCACCCATGGCAAATTCCATG-3' and antisense 5′-TCTAGACGGCAGGTCAGGTCCACC-3'. The resulting cDNAs were purified by High Pure PCR pruduct (Boehringer, Mannheim, Germany) according to the manufacturer's protocol. Then, probes were labelled with [^32^P]dCTP by random primer labelling. Northern hybridisation was performed according to standard protocols. The hybridised RNA was detected by autoradiography.

### Data analysis

Scanlyser software developed by Micheal Eisen at Lawrence Berkeley National Laboratory and GEArray analyser software (by SuperArray Inc.) were used to analyse the gene spots and process data. First X-ray film-recorded array images were converted into raw data files by using scanlyser software. The data files were then processed with GEArray analyser software. Background subtraction was performed normalising the data to the negative control (bacterial plasmid pUC18). The gene expression levels in the different samples were then normalised to the housekeeping gene expression level of each array. The results were expressed as relative amounts in arbitrary units. The data were presented as the mean±s.e.m. of two different experiments in duplicate.

### Western blot analysis

In a set of experiments, subconfluent glioblastoma cell lines grown in 60 mm plastic Petri dishes were starved for 24 h and then incubated in the presence of TRAIL (25 ng ml^−1^) for 12 and 24 h. In other experiments, cells grown in 60 mm plastic Petri dishes, starved for 24 h, were incubated in the presence of TRAIL (25 ng ml^−1^) or TNF*α* (25 ng ml^−1^) for 24 h, either alone or in combination. Cells were then lysed in NP-40 lysis buffer (50 mM HEPES, pH 7.6, 150 mM NaCl, 50 *μ*M NaF, 2 mM EDTA, 1 mM Na_3_VO_4_, 1% NP-40, 2 mM PMSF) and cellular extracts (30 *μ*g) were processed for SDS–PAGE electrophoresis and nitrocellulose membrane transfer. Membranes were incubated with a rabbit polyclonal anti-hVEGF (Santa Cruz Biotechnology, Santa Cruz, CA, USA), mouse monoclonal anti-hMMP-2 (Chemicon International Inc., Temecula, CA, USA), mouse monoclonal anti-hTIMP-2 (Santa Cruz Biotechnology) or goat polyclonal anti-hSPARC antibody (Santa Cruz Biotechnology). Secondary antibodies (Amersham Life Science, Buckinghamshire, UK) and a chemiluminescence kit (Amersham) were used for immunodetection. For validation of blot data, densitometric analysis was performed on immunoblots by using a KLB 2222-020 Ultra Scan XL laser densitometer at a wavelength of 633 nm. Western blot analysis was performed on samples from three separated experiments.

### ELISA test

A specific double-antibody ELISA (Oncogene Research Product, San Diego CA, USA) was used to determine the concentration of VEGF in the media of different glioblastoma cells treated with TRAIL (25 ng ml^−1^) for 24 h. The standard curve was generated using human recombinant VEGF_165_. The values of intra- and interassay variation were essentially within the range given by the manufacturer, that is, 3.5–6.5 and 5.0–8.5%, respectively. All values were assessed at least in quadruplicate.

### Viability assays

A172, U87MG and U373MG human glioblastoma cells were seeded at 1 × 10^3^ cells well^−1^ in 96-multiwell plates containing DMEM, with 10% FCS, penicillin and streptomycin. After 24 h, TRAIL was added at graded concentrations (range 12–200 ng ml^−1^) for 24 additional hours.

HUVECs were seeded at 1 × 10^3^ cells well^−1^ into 96-multiwell plates in M199 with ECGS, 20% FBS and heparin. After 24 h, medium was replaced with fresh complete medium containing graded concentrations of TRAIL (100, 200 and 400 ng ml^−1^), and incubated for 24 h.

At the end of all experiments, cells were stained with 0.5% crystal violet solution for 30 min, washed with bidistilled water and lysed in 10% acetic acid for 15 min. Optical density was read at 570 nm.

### *In vivo* experiments on angiogenesis

Male C57BL/6 mice, 6–8 weeks old, were purchased from Charles River (Calco, Italy). In National Cancer Research Institute's Animal Facility, investigators can work with laboratory animals with respect to the national current regulations regarding the protection of animals used for scientific purpose (D.L.vo 27/01/1992, no. 116) and research protocols were reviewed and approved by the Ethical Committee. All *in vivo* procedures met the standards required by the United Kingdom co-ordinating committee on cancer research (UKCCCR) guidelines. In addition, the animal study procedures were consistent and in accordance with the UKCCCR guidelines for the welfare of animals in experimental neoplasia (Workman *et al*, 1998).

The Matrigel sponge model of angiogenesis *in vivo* introduced by Passaniti *et al* (1992) and modified by Albini *et al* (1994, 1995) was used. VTH (50 ng ml^−1^ VEGF, 2 ng ml^−1^ TNF-*α* and heparin) alone or in combination with TRAIL was added to unpolymerised liquid Matrigel at 4°C, and the mixture brought to a final volume of 600 *μ*l. The Matrigel suspension was then injected slowly subcutaneously into the flanks of C57/bl6 male mice (six animals per group; housed in tight accordance to current GLP regulations and humane care; these procedures meet the standards required by the UKCCCR guidelines). In addition, the animal study procedures were consistent and in accordance with the UKCCCR guidelines for the welfare of animals in experimental neoplasia (Workman *et al*, 1998) with a cold syringe. At body temperature *in vivo*, the Matrigel quickly polymerises to form a solid gel. Different groups of animals were used for the different treatments. The control group was treated with vehicle alone; one group received TRAIL high dose (200 ng ml^−1^) with VTH in the Matrigel sponge, whereas another group received TRAIL low dose (100 ng ml^−1^) with VTH in the Matrigel sponge.

After 4 days, gels from all groups were collected and weighted. Samples were either minced and diluted in water to measure the haemoglobin content with a Drabkin reagent kit (Sigma) or processed for histology.

### Matrigel morphogenesis assay

Matrigel was thawed at 4°C in an ice/water bath, and 300 *μ*l well^−1^ were carefully added to a 24-microwell plate prechilled at −20°C using a cold pipette. Matrigel was allowed to polymerise for 30 min at 37°C. Once polymerisation had occurred, 70 000 HUVEC cells well^−1^ in 1 ml of complete medium were layered on the top of polymerised gel in the presence or absence of TRAIL at the concentrations indicated. The plates were then incubated at 37°C in a 5% CO_2_, humidified atmosphere. After 6 and 24 h, the wells were photographed with CCD optics and a digital analysis system (Image Pro Plus), respectively.

### Statistical analysis of results

Results were analysed by either one- or two-way analysis of variance (ANOVA), followed by Duncan's least significant difference test. Where appropriate, the Student's ‘*t*’-test was applied. Significance was admitted for a *P*-value <0.05.

## RESULTS

### Effects of TRAIL on human glioblastoma cell viability

**Figure 1:**
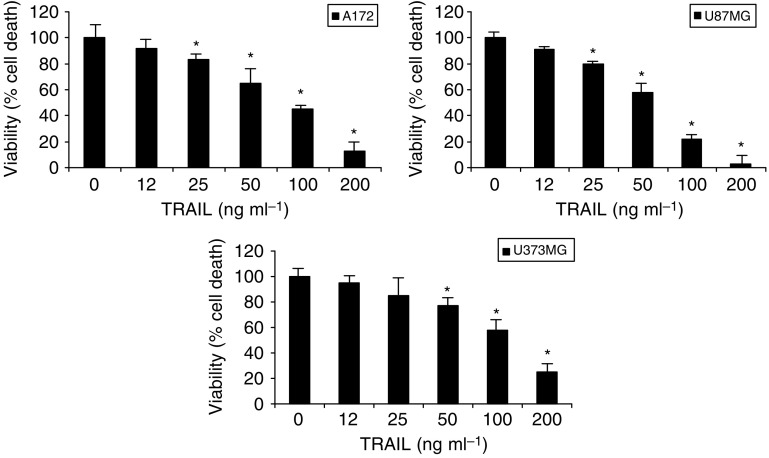
Concentration-related effects of TRAIL on the viability of the human glioblastoma cell lines A172, U87MG or U373MG. Vertical bars are means±s.e.m. All experiments were run three times in duplicate. ^*^P<0.05 compared to control (one-way ANOVA followed by Duncan's test).

We first assessed TRAIL cytotoxicity on the glioblastoma cell lines A172, U87MG and U373MG. All glioblastoma cells treated with graded concentrations of TRAIL showed significant decrease of viability, reaching a peak at a concentration of 100 ng ml^−1^. All cells underwent death after 24 h treatment. On the other hand, only about 25% of cells were killed after 24 h of treatment with TRAIL (25 ng ml^−1^) (Figure 1A, graphs 1, 2 and 3).

### Effects of TRAIL on human glioblastoma cell expression of genes related to angiogenesis, tissue remodelling and cell invasiveness

To study the effects of TRAIL on angiogenesis, we first investigated whether it could affect the expression of angiogenesis-related genes in glioblastoma cells. To do so, we performed multiple mRNA screening analysis in A172 cells treated with TRAIL.

The corresponding gene array analysis of A172 human glioblastoma cells treated with TRAIL showed that the response to TRAIL encompasses several angiogenesis-related genes.

**Figure 2:**
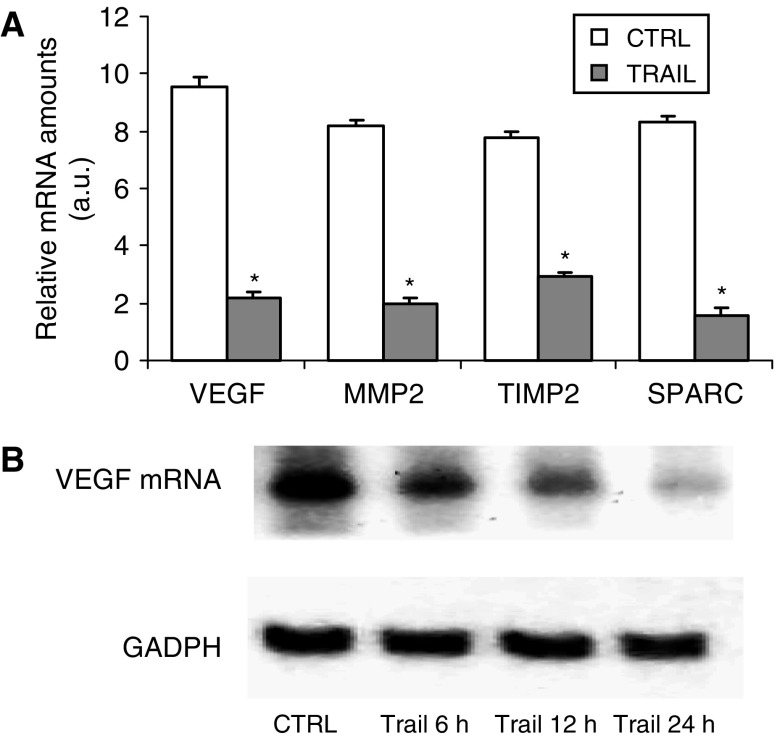
(**A**) Inhibition of VEGF, MMP-2, TIMP-2 or SPARC (open bars) mRNAs after exposure of A172 cells to TRAIL (closed bars), as revealed by cDNA microarray. mRNA was isolated from cells exposed for 24 h to 25 ng ml^−1^ of TRAIL. The results were expressed as relative amounts in arbitrary units. All data are the mean±s.e.m. from three different experiments run in duplicate. ^*^P<0.05 compared to control (one-way ANOVA followed by Duncan's test). (**B**) Northern blot analysis of the time-related inhibition of VEGF mRNA in A172 glioblastoma cells treated with 25 ng ml^−1^ of TRAIL. Quantitative analysis was performed normalising the sample *vs* GADPH mRNA levels. Time 0 was assumed as a control.

Particularly, the expression of the following genes was affected in A172 cells after 24 h of treatment with TRAIL: VEGF, the MMP-2, the gene encoding TIMP-2, a protein regulating the activity of MMP-2 and SPARC, a protein involved in glioblastoma invasiveness processes (Figure 2A).

### Effects of TRAIL upon the angiogenic potential of human glioblastoma cells: inhibitory effects of TRAIL on VEGF mRNA and protein

As VEGF expression was affected in A172 cells treated with TRAIL, we first attempted to measure changes in VEGF mRNA amounts by Northern blot analysis in cultures treated with TRAIL. The data obtained showed that VEGF mRNA was reduced after 6, 12 and 24 h of treatment with TRAIL. The effect of TRAIL was time-related and peak values were observed after 12 h of treatment (Figure 2B).

**Figure 3:**
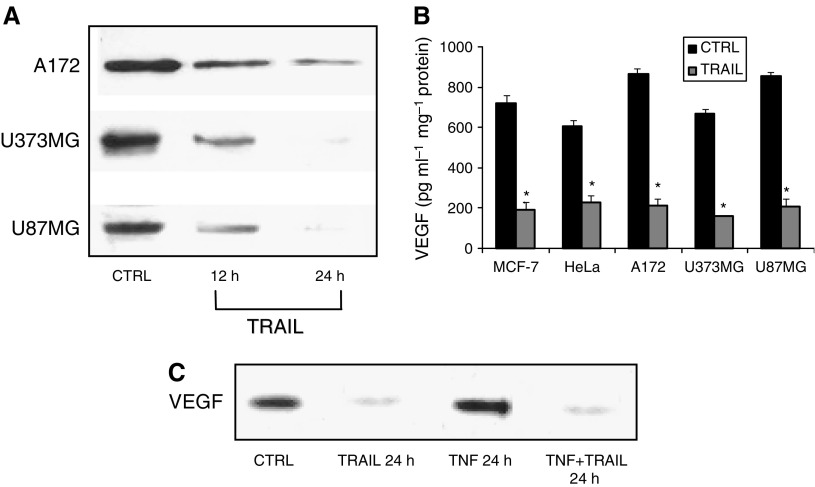
(**A**) Western blot analysis of the time course related to inhibition of the 21 kDa VEGF protein in A172, U373MG and U87MG glioblastoma cells treated with 25 ng ml^−1^ of TRAIL. Time zero was assumed as a control. (**B**) Amounts of VEGF released into the tissue culture media from either MCF-7, HeLa or A172, U373MG and U87MG glioblastoma cells incubated 24 h with TRAIL (25 ng ml^−1^). All data are the mean±s.e.m. from three different experiments run in duplicate. ^*^P<0.05 *vs* control (zero concentration; one-way ANOVA followed by Duncan's test). (**C**) Inhibitory effect of TRAIL upon the TNF-*α*-stimulated VEGF expression in A172 glioblastoma cells. TRAIL was added to cultures for 24 h at a concentration of 25 ng ml^−1^. TNF-*α* was used at a concentration of 25 ng ml^−1^.

Secondly, Western blot analysis showed a reduction in the amounts of VEGF protein in the three glioblastoma cell lines treated 24 h with TRAIL (Figure 3A).

In addition, as the VEGF synthesised could not have necessarily been released in the *milieu*, we measured VEGF levels in the culture media of the same samples of glioblastoma cells and observed that it was actually released in substantial amounts (Figure 3B).

Nevertheless, the effects of TRAIL described so far appeared related to its capability of interfering with basal levels of VEGF protein expressed by A172, U87MG and U373MG cells. As it is known that angiogenesis is the result of a complex interplay of an array of different factors, we studied whether such inhibitory effect of TRAIL could interfere with proangiogenic factor-stimulated VEGF expression.

Thus, we first induced high VEGF expression by treating A172 cells with TNF-*α*, a known inducer of VEGF expression (Ferrer *et al*, 1997). However, VEGF expression did not increase in the presence of TNF-*α* in A172 cells preincubated with TRAIL. The effect of the latter was time-dependent and reached its peak after 24 h (Figure 3C).

### Effects of TRAIL on normal human endothelial cells: TRAIL affects VEGF synthesis capacity and mitogenesis in HUVEC cells

Endothelial cells respond to VEGF with mitogenesis, followed by the organisation of cells in new vessels. VEGF may either be released in the *milieu* either by tumour cells or by endothelial cells themselves. In order to verify whether the effects of TRAIL on VEGF production by tumour cells are actually concurring with a general antiangiogenic objective, the angiogenesis-related gene array analysis was also performed in HUVEC.

**Figure 4:**
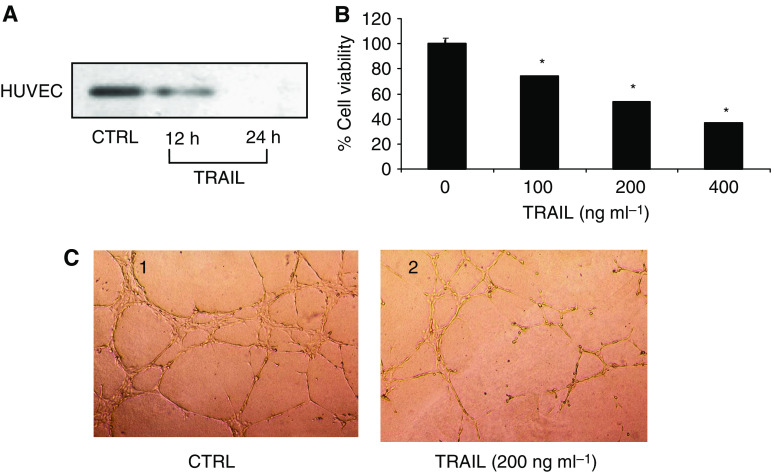
(**A**) Western blot analysis of the time-dependent effect of TRAIL (100 ng ml^−1^) upon the expression of the 21 kDa VEGF protein in HUVEC cells. (**B**) Concentration-dependent viability of HUVEC cultures treated 24 h with graded concentrations of TRAIL. All data are the mean±s.e.m. from three different experiments run in duplicate. ^*^P<0.05 *vs* control (zero concentration; one-way ANOVA followed by Duncan's test). (**C**) Matrigel morphogenesis assay *in vitro*. Tube formation was evaluated in HUVEC cells grown in matrigel and either untreated (control, 1) or treated for 24 h with 200 ng ml^−1^ of TRAIL (2).

VEGF protein expression in HUVEC, which expresses both TRAIL receptors DR4 and DR5 mRNAs and proteins, was reduced after 24 h of treatment with TRAIL (Figure 4A), an effect paralleled by reduced proliferation of endothelial cells (Figure 4B).

#### TRAIL inhibits vessel formation *in vitro* and *in vivo*

To assess whether the antimitogenic effect of TRAIL on HUVEC encompasses actual blood vessel formation, we tested the effects of TRAIL in appropriate models, such as tube formation in matrigel *in vitro* (matrigel morphogenesis assay), as well as vessel formation in matrigel sponges containing a proangiogenic cocktail implanted subcutaneously *in vivo* in the mouse (inhibition of angiogenesis *in vivo*, according to the UKCCCR guidelines; Workman *et al*, 1998).

In the matrigel morphogenesis assay, formation of the typical cellular network occurred 6 h after plating (Figure 4C, photograph 1); TRAIL had concentration-dependent inhibitory effect on the morphogenesis of HUVEC cells and formation of capillary-like structures, which peaked at 200 ng ml^−1^ (Figure 4C, photograph 2).

**Figure 5:**
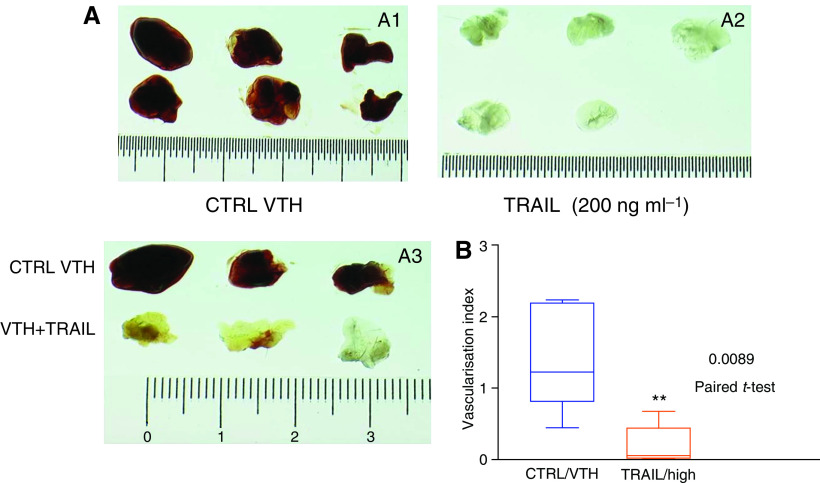
Matrigel angiogenesis assay *in vivo*. The figure illustrates the differences in size (cm) of the vascularised areas developed locally *in vivo* after stimulation with different substances. All *in vivo* procedures were performed in accordance to the standards required by the UKCCCR guidelines for the welfare of animals in experimental neoplasia (Workman *et al*, 1998). (**A**) Effect of TRAIL upon vessel formation induced by matrigel sponges containing a proangiogenic cocktail implanted subcutaneously *in vivo* in the mouse. Either a proangiogenic cocktail (VTH alone, the six samples shown in A1), TRAIL at a concentration of 200 ng ml^−1^ (TRAIL alone, the six samples shown in A2), or a combination of both (in A3: VTH alone, the three samples shown in the upper row; VTH+TRAIL, the three samples in the lower row) was added to matrigel. (**B**) Vascularisation index calculated on the basis of the inhibitory effects of TRAIL measured in the matrigel angiogenesis assay *in vivo*. ^*^P<0.009 (Student's ‘*t*’-test for paired differences).

The effects of TRAIL on angiogenesis-associated endothelial cell functions observed *in vitro* were confirmed *in vivo* in the Matrigel angiogenesis assay. Matrigel suspensions containing a proangiogenic cocktail with VEGF (50 ng ml^−1^), TNF-*α* (2 ng ml^−1^) and heparin (VHT) were injected subcutaneously into mice. The presence of VTH in the Matrigel sponges promoted a haemorrhagic vascularisation of the gels within 4 days (Figure 5A, photograph 1). Histological examination confirmed the absence of vascularisation in the samples treated with TRAIL alone (Figure 5A, photograph 2), or the blunted angiogenesis also in the presence of the angiogenic cocktail VTH (Figure 5A, photograph 3). Quantification of the extent of angiogenesis by haemoglobin content measurement showed that TRAIL (200 ng ml^−1^) significantly (*P*<0.0089; paired *t*-test) reduced the angiogenic response if compared to the positive control (Figure 5B).

### Effects of TRAIL on the expression of the MMP-2 and its inhibitor TIMP-2, and the invasiveness regulator protein SPARC in human glioblastoma cell lines

The angiogenic process appears closely related to the capacity of the tumour itself to express local invasiveness. Interestingly, gene array analysis showed that mRNA levels of both metalloproteinase MMP-2 and its inhibitor TIMP-2 were reduced in A172, U87MG and U373MG cells treated for 24 h with TRAIL.

**Figure 6:**
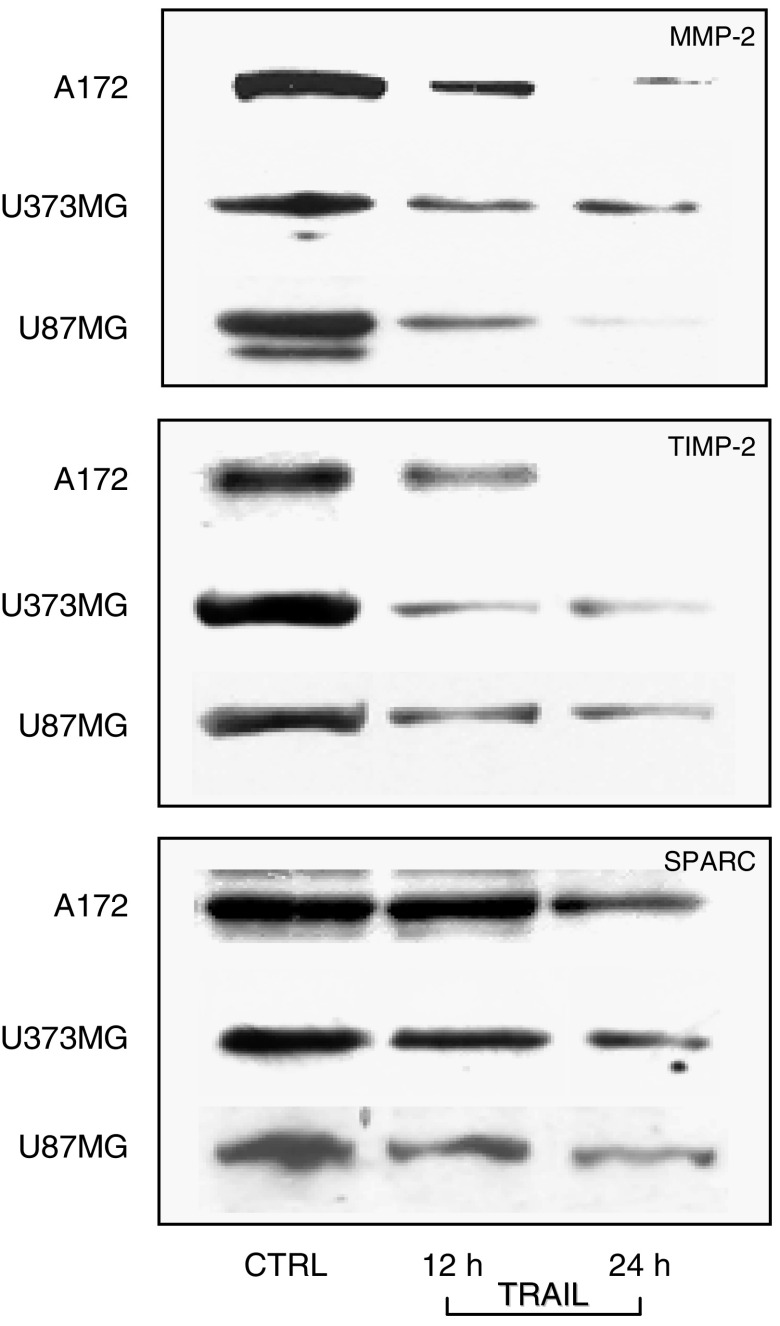
Western blot analysis of the inhibitory, time-dependent effects of TRAIL (25 ng ml^−1^) upon the expression of MMP-2 (upper panel), its inhibitor TIMP-2 (middle panel) and SPARC (lower panel) proteins in cultured A172, U373MG and U87MG human glioblastoma cell lines.

Thus, we first assessed the amounts of intracellular MMP-2 by Western blot analysis of proteins. The results indicated that TRAIL induces a time-dependent decrease in intracellular levels of the enzyme. Peak of TRAIL effect occurred at 24 h (Figure 6, upper bands).

Along with the MMP-2, we studied the expression of its protein inhibitor TIMP-2, whose expression was also decreased in the gene array analysis.

Western blot analysis of TIMP-2 in A172, U87MG and U373MG cells treated with TRAIL resulted in decreased levels of TIMP-2 protein. The effect of TRAIL was time-dependent. Peak of the inhibitory effect was at 24 h (Figure 6, middle bands).

The protein SPARC has been regarded as a promoter of local invasiveness of glioblastoma. As we have shown that TRAIL decreases angiogenesis as well as remodelling factors, we attempted to answer the question whether factors controlling invasiveness could also be affected by TRAIL.

Interestingly, gene array analysis of A172 cells treated with TRAIL showed that SPARC mRNA was reduced in comparison with untreated cells.

On the same line, Western blot analysis data showed decreased amounts of SPARC protein in A172, U87MG and U373MG cells treated with TRAIL, with a maximal effect at 24 h (Figure 6, lower bands).

## DISCUSSION

We showed that TRAIL, a typical proapoptotic peptide molecule (Sheridan *et al*, 1997; Almasan and Ashkenazi, 2003), is a potent inhibitor of angiogenesis-, tissue remodelling- or invasiveness-related factors in human glioblastoma cells. In gene array experiments, we observed that the treatment of A172 human glioblastoma cells with TRAIL reduced VEGF mRNA expression. In addition, expression of the tissue remodelling-related gene MMP-2 and its regulator TIMP-2 was also decreased, along with the expression of the invasiveness-related protein SPARC. Northern and Western blot analyses confirmed gene array data, by showing a decrease in VEGF mRNA in A172 cells, and the corresponding protein in A172, U87MG and U373MG cells treated with TRAIL.

Human glioblastoma is a richly vascularised tumour (Zagzag *et al*, 2000) that expresses and actively releases in the vicine tissues substantial amounts of factors promoting new vessel growth (Guerin and Laterra, 1997). As a counterproof of such biological attitude of glioblastoma, we also found that VEGF is actively released by A172, U87MG and U373MG glioblastoma cells in the tissue culture media.

Thus, from these data, it is plausible to hypothesise that VEGF released in the *milieu* probably reaches biologically significant concentrations, which, in light of its proangiogenic effects (Ferrara *et al*, 2003), are functional to maintain blood vessel formation aimed to fuel tumour enlargement.

Not only were the antiangiogenic effects of TRAIL on glioblastoma cells related to inhibition of VEGF release by tumour cells, but these were also extended to endothelial cells.

In fact, TRAIL was able to reduce the expression of VEGF protein in HUVECs. VEGF produced and released by endothelial cells is regarded as an ancillary mechanism supporting their own survival and proliferation in an autocrine fashion (Villegas *et al*, 2005), as it has been described, for instance, in ischaemic tissues (Seghezzi *et al*, 1998).

In addition to its anti-VEGF effect in HUVECs, TRAIL reduced not only HUVECs' viability but also their capability of organisation in primitive vessels, as shown by data indicating TRAIL-dependent inhibition of vessel formation *in vitro* and *in vivo*.

In support of these results, Gochuico *et al* (2000) described activation of an intracellular death programme, followed by apoptosis, in endothelial cells treated with concentrations of TRAIL in the range of hundreds of nanograms per millilitre, which have been shown to induce apoptosis in a variety of cells (Puduvalli *et al*, 2005; Shiraki *et al*, 2005). Thus, the apparent discrepancy of ours with other data demonstrating a mitogenic effect of TRAIL upon human endothelial cells (Secchiero *et al*, 2004) could be explained on the basis of the significantly lower (about 10-fold; 10 ng ml^−1^) concentrations used.

In fact, it has been demonstrated that low concentrations of TRAIL activate signal-transduction pathways related, respectively, to ERKs and Akt, the latter depending upon PI3K activation (Secchiero *et al*, 2003), whereas p38K is not activated. Besides, TRAIL at a concentration of 100 ng ml^−1^ induces apoptosis in combination with the PI3K inhibitor LY294002 in human vascular endothelial cells through activation of the extrinsic pathway, causing progressive cleavage of caspase-8 and caspase-3 with concurrent reduction of the antiapoptotic gene bcl-2 and early loss of the short form of the cellular FLIP (Alladina *et al*, 2005).

At any rate, we were also able to show the antiangiogenic effect of TRAIL in various *in vitro* models. To strenghten the hypothesis that TRAIL possessed antiangiogenic properties, we have also shown that it displays antagonistic activity upon proangiogenic cocktail-stimulated vessel formation *in vivo* in the mouse.

Evidence suggests that the angiogenic process is associated with a complex network of cellular and molecular events related to tissue remodelling (Sounni *et al*, 2002) and tumour tissue invasiveness (Nakada *et al*, 2003).

The human glioblastoma cell lines A172, U87MG and U373MG expressed substantial amounts of the MMP-2 (Chintala *et al*, 1999), which were decreased after treatment with TRAIL.

Tissue metalloproteinases, such as MMP-2, appear specifically involved in tissue remodelling. In fact, it has been shown that their expression is significantly increased in inflammation (Shamamian *et al*, 2001) and in tumour invasiveness and metastatisation (Turck *et al*, 1996) processes. Metalloproteinases are in turn regulated by inhibitory molecules (Goldman and Shalev, 2004); the balance between the two molecular families being critical for control of proteolysis (Lu *et al*, 2004).

Interestingly, the A172, U87MG and U373MG glioblastoma cell lines expressed high levels of the TIMP-2 metalloproteinase inhibitor and TRAIL was able to reduce such expression. Although divergent data are available regarding relationships between the expression of MMPS and the inhibitory TIMP protein family in gliomas, a number of reports claim that, along with that of MMP, TIMP overexpression is closely related to the degree of malignancy (Nakano *et al*, 1995; Lampert *et al*, 1998).

Overall, the bulk of data demonstrate that an altered ratio between TIMP-2 and MMP-2 may be related to glioblastoma invasiveness (Kachra *et al*, 1999).

It could be reasoned that the inhibitory effect of TRAIL upon the metalloproteinase system is added to its antiangiogenic effects reported earlier. In fact, factors controlling tumour invasiveness act in a concerted way with those related to both angiogenesis and tissue remodelling (Finn *et al*, 1997). Interestingly, gene array data from cells treated with TRAIL showed decreased expression of the invasiveness inhibitor protein SPARC. Although the latter normally inhibits cell invasiveness processes (Hasselaar and Sage, 1992), it displays opposite effects in tumour cells (Briggs *et al*, 2002), including glioblastoma (Vajkoczy *et al*, 2000). For this reason, SPARC has been regarded as an enhancer of the capability of glioblastomas to localise in multiple sites of the brain (Schultz *et al*, 2002).

In summary, TRAIL is a potent inhibitor of the orchestra of factors controlling angiogenesis, invasiveness and tissue remodelling in various human glioblastoma cell lines. The antiangiogenic effects of TRAIL include inhibition of VEGF release from tumour cells, as well as an antimitogenic effect upon endothelial cells. In addition, TRAIL potently suppresses the expression of tissue remodelling factors, such as MMP-2 and TIMP-2, as well as of those promoting glioblastoma invasiveness, such as SPARC.

In conclusion, the TRAIL system could be regarded as a potential target for novel glioblastoma therapy aimed at disruption of the *ensemble* of factors promoting angiogenesis, tissue remodelling and tumour invasiveness.
